# The SKP2–Substrate Axis in Hematological Malignancies: Mechanisms of Oncogenesis and Opportunities for Targeted Therapy

**DOI:** 10.3390/cells15191796

**Published:** 2026-09-30

**Authors:** Yueying Zhang, Omar Faruq, Mariusz Shrestha, Shamila D. Alipoor, Hong Chang

**Affiliations:** 1 Laboratory Medicine Program, Department of Laboratory Hematology, University Health Network, Toronto, ON M5G 2C4, Canada; 2 Department of Pathophysiology, College of Clinical and Basic Medicine, Shandong First Medical University, Jinan 250017, China; 3 Department of Laboratory Medicine and Pathobiology, University of Toronto, Toronto, ON M5S 1A8, Canada; 4 Department of Biomedical and Clinical Sciences, Linköping University, SE-581 83 Linköping, Sweden

**Keywords:** SKP2, SCF complex, ubiquitination, hematological malignancies, targeted therapy, prognostic marker

## Abstract

S-phase kinase-associated protein 2 (SKP2), a key substrate-recognition subunit of the Skp1-Cul1-F-box (SCF) E3 ubiquitin ligase complex, promotes the ubiquitin–proteasome-mediated degradation of tumor suppressors such as p27^Kip1^ and p21^(Cip1/Waf1)^. Through these activities, SKP2 regulates critical cellular processes, including cell cycle progression, apoptosis, metabolic reprogramming, maintenance of cancer stem cell stemness, and contributes to the pathogenesis, therapeutic resistance, and clinical progression of hematological malignancies. Recent advances in protein degradation technologies and regulatory network analysis have highlighted SKP2 and its substrate interactions as promising targets for precision therapies. In this review, we examine the biological functions of SKP2 and regulatory mechanisms governing its substrate spectrum across hematological malignancies, including acute myeloid leukemia, chronic myeloid leukemia, B/NK/T-cell lymphoma, and multiple myeloma. We further review currently available SKP2-targeted therapeutic strategies, including small-molecule inhibitors, molecular glue degraders, and proteolysis-targeting chimeras (PROTACs), and discuss SKP2-related prognostic biomarkers in hematological malignancies. Finally, we outline the key challenges and future directions to guide the development of SKP2-centered therapeutic approaches.

## 1. Introduction

Hematological malignancies are a group of heterogeneous cancers and remain a major cause of cancer-related morbidity and mortality worldwide [1,2]. The development of targeted therapy has become a key direction for improving patient prognosis. The ubiquitin–proteasome system (UPS) is a core pathway for selective protein degradation in eukaryotic cells [3,4]. Through selective ubiquitination and proteasomal degradation of regulatory proteins, the UPS participates in various physiological processes including cell cycle, signal transduction, and DNA damage repair [3,4,5]. Dysregulation of the UPS can lead to the accumulation of oncogenic proteins or the abnormal degradation of tumor suppressor proteins, which drive tumorigenesis and disease progression [6,7,8,9]. SKP2 was first identified in 1995 as a key F-box protein in the Skp1-Cul1-F-box E3 ubiquitin ligase complex [10]. Through its C-terminal leucine-rich repeat domain and in cooperation with the adaptor protein Cks1, SKP2 recognizes phosphorylated cell-cycle substrates, thereby promoting their ubiquitination and proteasomal degradation [11,12]. In hematological malignancies, SKP2 is frequently overexpressed, leading to accelerated degradation of tumor suppressors such as p27^Kip1^ and p21^(Cip1/Waf1)^. Consequently, SKP2 enhances malignant cell proliferation, suppresses apoptosis and promotes therapeutic resistance in hematological malignancies [13,14,15,16,17].

Notably, SKP2 expression exhibits considerable heterogeneity across hematological malignancies and is closely linked to disease aggressiveness and clinical prognosis [13,14,18,19]. Indolent lymphomas, including small lymphocytic lymphoma and follicular lymphoma, generally display low SKP2 immunoreactivity. By contrast, aggressive B-cell neoplasms such as mantle cell lymphoma (MCL) and diffuse large B-cell lymphoma (DLBCL) show upregulation of SKP2 protein levels, which correlate strongly with enhanced proliferative index, as reflected by the elevated Ki-67 labeling [13,15,16]. Importantly, high SKP2 protein expression predicts inferior clinical outcomes, as demonstrated by shorter progression-free survival (PFS), reduced overall survival (OS) and disease-free survival (DFS) in MCL, DLBCL, acute myeloid leukemia (AML) and extranodal NK/T-cell lymphoma (ENKTL) [14,18,19]. Beyond driving intrinsic tumor progression, SKP2 also fuels disease aggressiveness by mediating therapeutic resistance. In multiple myeloma (MM), concurrent SKP2 protein upregulation and p27 protein reduction are linked to bortezomib resistance and inferior clinical outcomes [20]. Furthermore, retrospective patient-cohort analyses indicate that elevated SKP2 protein expression is associated with high-risk disease features [14]. However, SKP2 remains an investigational biomarker that has not been prospectively validated for clinical risk-stratification or therapeutic decision-making. Taken together, these findings support SKP2 as a biomarker of disease aggressiveness, a prognostic indicator, a potential guide to treatment decisions, and an intriguing target in hematological malignancies [18,19,20,21].

Recent preclinical and translational studies have substantially advanced our understanding of SKP2 biology and accelerated the development of SKP2-targeted therapeutic strategies. Until recently, SKP2 was considered undruggable, largely owing to the absence of classic ligand-binding pockets and extensive protein–protein interactions that hinder specific small-molecule intervention [22]. To overcome this limitation, two targeted protein degradation modalities have been developed. Molecular glues are small molecules that induce neo-protein–protein interactions (neo-PPIs) to trigger substrate ubiquitination and subsequent proteasomal degradation. For instance, the SKPer1 (XMU-MP-8) molecular glue degrader, first reported in 2025, displays robust antitumor efficacy accompanied by favorable in vivo safety profiles in preclinical hematological malignancy models [23]. Proteolysis-targeting chimeras (PROTACs) are bifunctional hybrid molecules consisting of a target protein-binding ligand, an E3 ubiquitin ligase recruiter, and a chemical linker. These agents redirect the endogenous ubiquitin–proteasome system to specifically eliminate oncogenic proteins. The fast-evolving pipeline of SKP2-targeted molecular glues and PROTAC degraders enables robust pharmacological depletion of SKP2 protein, offering promising therapeutic strategies for SKP2-driven hematological malignancies [24,25,26]. This review summarizes the role of SKP2 and its substrate targets in hematological malignancies, highlights the advances in SKP2-targeted therapies, and discusses the challenges and future prospects for their clinical translation.

## 2. Core Molecular Mechanisms and Oncogenic Functions of SKP2

**Figure 1:**
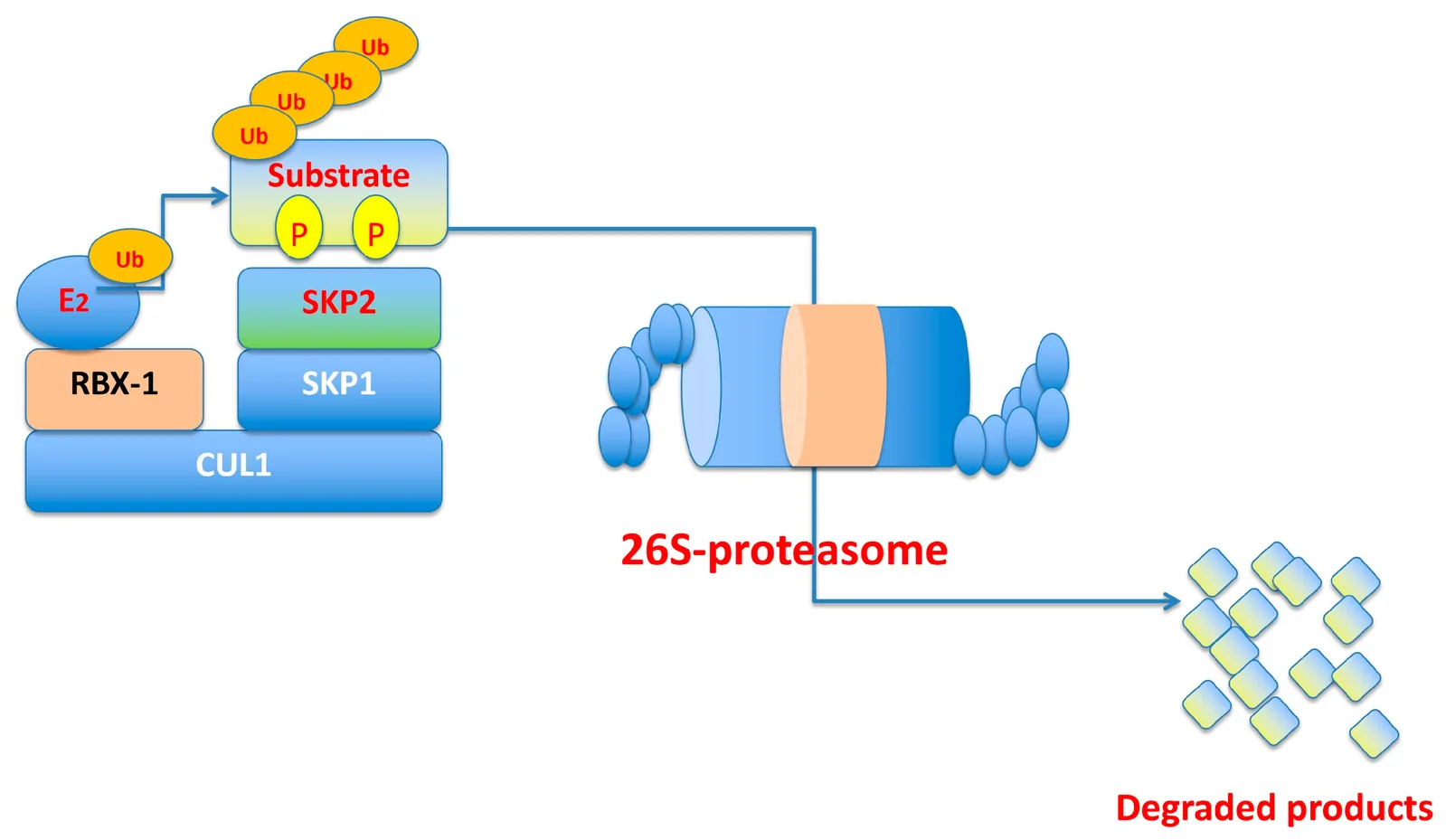
Schematic diagram of the core canonical degradative function of SKP2 within the SCF E3-ligase complex. Note that the adaptor protein Cks1, which facilitates recognition of phosphorylated substrates by SKP2, is not depicted in this simplified diagram. SKP2 can also mediate non-degradative K63-linked ubiquitination of target proteins, as described in the main text.

SKP2 primarily functions as the substrate-recognition subunit of the SCF complex (Figure 1). It forms a stable complex with Skp1, Cul1, and Rbx1 subunits, to specifically recognize and bind phosphorylated target proteins and recruit E2 ubiquitin-conjugating enzymes that mediate polyubiquitination, and subsequent degradation of target proteins by the 26S proteasome [10,11]. The expression level of SKP2 is cell cycle-dependent, remaining low in the G1 phase and significantly increased in the S and G2/M phases. It promotes the G1-S phase transition by degrading cell cycle inhibitors, serving as a key positive regulator of cell cycle progression [27].

Beyond the classic cell cycle regulatory function, emerging evidence indicates that SKP2 also participates in regulating various biological processes such as tumor metabolic reprogramming including the Warburg effect [28], maintenance of cancer stem cell stemness [29,30,31], and regulation of tumor immune microenvironment [32,33]. The diversity of its functions is closely related to its broad spectrum of substrate proteins [33].

## 3. Key SKP2 Interacting Molecules and Context-Dependent Oncogenic Functions in Various Hematological Malignancies

As a core F-box subunit of the SCF E3 ubiquitin ligase complex, SKP2 engages in functionally distinct molecular interactions. It acts primarily by mediating K48-linked polyubiquitination and proteasomal degradation of direct proteolytic substrates [5,33]. In addition, SKP2 can mediate non-degradative ubiquitination to alter target-protein activity or localization without triggering degradation [33]. A third group of proteins function as upstream regulators or functional binding partners: they control SKP2 expression or activity but are not themselves ubiquitinated by the SCF–SKP2 complex [34].

Many direct SKP2 substrates are tumor suppressors present in hematological malignancies as well as numerous other cancer types. By mediating the degradation of these substrates, SKP2 eliminates their tumor-suppressive functions and facilitates tumor progression [33,34]. Distinct classes of SKP2-interacting molecules exert diverse biological processes that are co-opted during leukemogenesis and lymphomagenesis, and their coordinated loss of function forms the core of the SKP2-orchestrated oncogenic signaling axis summarized in Table 1.

Below, we summarize the substrate-specific functions of SKP2 and its distinct oncogenic roles in the major types of hematological malignancies.

### 3.1. Acute Myeloid Leukemia

AML is a highly heterogeneous hematologic neoplasm characterized by the clonal expansion of immature myeloid blasts in the bone marrow and peripheral blood, leading to impaired hematopoiesis, bone marrow failure, and life-threatening complications including severe infections and bleeding [42,43]. AML arises from acquired somatic mutations in hematopoietic stem and progenitor cells (HSPCs), which block myeloid differentiation and drive uncontrolled malignant proliferation [44].

SKP2 maintains physiological hematopoietic stem cell (HSC) homeostasis by proteasomal degradation of the cyclin-dependent kinase inhibitors (CDKIs), p27^Kip1^ and p57^Kip2^ [44]. CDKIs serve as negative regulators of cell-cycle progression that restrict G1/S transition, maintain HSC quiescence and regulate stress-induced hematopoietic regeneration [44]. The targeted degradation of CDKIs by SKP2 is a major driver of cell-cycle progression in AML [35]. Furthermore, elevated SKP2 activity reduces CDKIs levels, thereby driving not only uncontrolled proliferation but also the maintenance of leukemia stem cells [35]. An elevated SKP2–CKS1 axis activity contributes to AML pathogenesis. Importantly, pharmacological inhibition of the CKS1-SKP2 axis can deplete AML leukemic stem cells (LSCs) while largely sparing normal HSCs [45].

Mechanistically, SKP2 drives malignant proliferation in bulk AML cells by targeting three major CDKIs, p27^Kip1^, p21^(Cip1/Waf1)^, and p57^Kip2^, for degradation, thereby releasing G1 cell-cycle arrest and enabling S-phase entry [34]. In primary AML, quiescence enforced by CDKIs preserves LSC self-renewal and therapy resistance, whereas exit from G0 quiescence renders LSCs vulnerable to elimination [46]. Important insights regarding SKP2-dependent control of stem-cell quiescence are largely derived from studies in normal HSCs [47]. Preclinical evidence further supports that SKP2–CKS1-mediated proteostasis governs AML–LSC fate [37,45,47]. In LSC-enriched AML pre-clinical models, SKP2 contributes to sustaining stem-associated phenotypes via multiple non-mutually exclusive mechanisms, including disruption of CDKI-dependent quiescence control, cell-cycle recruitment of dormant LSCs, stabilization of telomerase reverse transcriptase (TERT) [37], and maintenance of MYC-driven oncogenic transcriptional programs [47].

First, SKP2-mediated degradation of p27^Kip1^, p21^(Cip1/Waf1)^ and p57^Kip2^ not only drives proliferation but also prevents LSC entry into G0 quiescence, a state that is linked to differentiation and loss of stemness in LSCs [47]. Second, SKP2 stabilizes telomerase reverse transcriptase (TERT) and sustains c-MYC protein abundance in malignant hematopoietic cells, reinforcing self-renewal and drug resistance [29,37]. SKP2–CKS1 inhibition reduces TERT, induces senescence, and sensitizes LSC-enriched cell populations to chemotherapy [37,45].

Thus, SKP2–CKS1 signaling is not just a cell-cycle driver but a key modulator of stemness phenotypes in AML–LSC preclinical models. Targeting SKP2–CKS1 signaling can disrupt LSC proteostasis, drive cell-cycle recruitment of dormant LSCs, and impair self-renewal features, highlighting its therapeutic potential against chemotherapy-resistant AML stem-cells [17,36,37,45,47].

Under steady-state conditions, SKP2 deletion enhances quiescence and expands the HSC pool, whereas under stress conditions, it impairs the ability of HSCs to rapidly enter the cell cycle [34]. Leukemic stem cells typically arise from dysregulated HSCs, and SKP2’s cell cycle regulatory functions are critical for AML malignant transformation and pathogenesis [34].

Beyond its pan-AML cell-cycle and stemness functions, SKP2 executes subtype-specific oncogenic programs in acute promyelocytic leukemia (APL), a subtype of AML [39]. APL is predominantly driven by the PML–RARα fusion protein as well as dysregulated JunB expression. As a critical pathogenic regulator in APL, SKP2 promotes disease progression through two major mechanisms. First, SKP2 stabilizes the PML–RARα fusion protein and prevents its degradation, thus sustaining its oncogenic activity [39]. Unlike most canonical SKP2 substrates that undergo proteasomal degradation upon ubiquitination, SKP2-mediated ubiquitination confers protein stability rather than degradation to the PML-RARα oncofusion protein. Furthermore, SKP2 downregulates JunB expression, impairing the differentiation of APL blasts [39]. These effects highlight SKP2 as a critical contributor to APL pathogenesis and a potential therapeutic target.

### 3.2. Chronic Myeloid Leukemia (CML)

The primary oncogenic driver of CML is the BCR-ABL fusion protein, which transcriptionally upregulates SKP2 expression by activating the PI3K/Akt/CREB signaling pathway, accelerating p27^Kip1^ degradation and promoting cell cycle progression [40]. Genetic deletion of SKP2 markedly alleviates BCR-ABL-driven myeloproliferative disease in vivo and prolongs the survival of affected mice. Loss of SKP2 also leads to elevated nuclear p27^Kip1^ in SKP2^−/−^ leukemic cells. These findings indicate that SKP2 is a critical mediator of BCR-ABL-induced leukemogenesis [41].

SKP2 is significantly overexpressed in patients with CML and is associated with a poor response to imatinib therapy, and may serve as an important prognostic predictor in CML [48]. Targeting SKP2 exhibits favorable anti-leukemic effects. Specifically, diosmetin suppresses CML cell proliferation by simultaneously downregulating SKP2 expression and inhibiting BCR-ABL activity [49]. Meanwhile, bortezomib induces apoptosis in CML cells by reducing SKP2 levels and increasing the accumulation of its downstream substrate p27^Kip1^, providing mechanistic evidence that the therapeutic effect of proteasome inhibitors in CML is mediated by SKP2 [50].

Although tyrosine kinase inhibitors (TKIs) that target BCR-ABL, such as imatinib are the first-line clinical therapy for CML, acquired drug resistance remains a major challenge that urgently needs to be addressed. Inhibition of SKP2 can significantly enhance the sensitivity of CML cells to imatinib by inducing p27^Kip1^ accumulation, and effectively reverse TKI resistance [51]. These findings strongly indicate that targeting SKP2 represents a promising therapeutic strategy to overcome TKI resistance in CML, and combined treatment with SKP2 inhibitors and TKIs may be an effective approach for CML.

### 3.3. B-Cell Acute Lymphoblastic Leukemia (B-ALL)

SKP2 upregulation in BCP-ALL is predominantly driven by hyperactivated PI3K/AKT signaling and other leukemic oncogenic cascades [34], and carries clear clinical relevance. Retrospective analyses of primary patient specimens reveal frequent SKP2 overexpression, which correlates with enhanced blast proliferation and intrinsic resistance to standard chemotherapies [34]. High SKP2 expression is associated with chemoresistance in BCP-ALL. SKP2-driven degradation of p21^(Cip1/Waf1)^ contributes to doxorubicin resistance [52,53]. Pharmacological inhibition of SKP2 combined with doxorubicin exhibited synergistic anti-leukemic effects. It led to p21^(Cip1/Waf1)^ accumulation, impaired DNA damage repair, and significantly enhanced doxorubicin-induced cell apoptosis [52,53]. Thus, suppression of SKP2 alleviates chemoresistance and potentiates chemotherapy-induced cytotoxicity. Selective SKP2 inhibitors induce robust caspase-dependent apoptotic death in BCP-ALL single-agent treatment, and exert synergistic cytotoxic effects when combined with doxorubicin [52]. Taken together, accumulating preclinical and clinical studies consistently identify SKP2 as a promising therapeutic target for BCP-ALL [34,52,53].

To systematically compare the conserved and divergent oncogenic functions of SKP2 across all hematological malignancies, Table 2 summarizes the key molecular mechanisms, malignant cellular phenotypes, and corresponding supporting references for each disease entity.

### 3.4. T-Cell Acute Lymphoblastic Leukemia (T-ALL) 

SKP2 functions as a critical oncogenic driver across T-cell acute lymphoblastic leukemia via conserved cell cycle regulatory cascades. The upstream signaling pathways that trigger SKP2 overexpression differ markedly between T-ALL and B-cell precursor (BCP-ALL) subtypes. In T-ALL, oncogenic NOTCH1 signaling is the dominant upstream regulator of SKP2. Activated NOTCH1 intracellular domain (NICD) directly binds to the SKP2 promoter and transcriptionally upregulates SKP2 expression, which in turn promotes the ubiquitination and degradation of p27^Kip1^, driving accelerated G1/S transition and leukemic cell proliferation [54]. Functional verification in vivo demonstrates that genetic ablation or pharmacological inhibition of SKP2 robustly suppresses leukemogenesis in T-ALL xenograft and primary murine models, with minimal interference on thymic T-cell development, highlighting the selective therapeutic window of SKP2-targeted intervention [55]. Collectively, these in vitro and in vivo data establish a canonical NOTCH1–SKP2–p27^KIP1^ signaling axis that maintains malignant progression in T-ALL [54,55].

### 3.5. B-Cell Lineage Lymphoma

SKP2 acts as a crucial oncogenic regulator in B-cell lymphoma. In DLBCL, 39.6% of patients have high SKP2 expression, which is closely associated with high Ki-67 expression and germinal center subtype, and significantly shortened overall survival (OS) [56]. Unlike the significant negative correlation between SKP2 and p27^Kip1^ in low-grade lymphoma, no obvious correlation between SKP2 expression and p27 was observed in DLBCL [56,58].

### 3.6. Multiple Myeloma

MM is a malignant proliferative disease of plasma cells, characterized by progressive genomic evolution, metabolic reprogramming, and the frequent emergence of therapy-resistant subclones—key features that contribute to disease recurrence and poor clinical outcomes [57,59,60]. Increasing evidence demonstrates that SKP2 expression rises during disease progression, correlating with higher tumor burden and poor survival [17,20,21,55]. Mechanistically, SKP2 promotes degradation of p27^Kip1^, promotes c-MYC-driven transcriptional programs, and intersects with proteasome-dependent survival pathways, to reinforce proliferation and stress tolerance [17,20,21,55].

Proteasome inhibitors represented by bortezomib constitute the first-line therapeutic agents for MM and have significantly improved patient survival. However, acquired drug resistance remains a major clinical challenge that limits their long-term therapeutic efficacy. It has been reported that bortezomib induces apoptosis in MM cells by downregulating SKP2 expression and promoting p27^Kip1^ accumulation. Moreover, retrospective analysis in MM patients shows that low SKP2 expression exhibits a higher bortezomib response rate [20]. This observation suggests that SKP2 may have predictive value for proteasome inhibitor sensitivity. However, SKP2 has not been validated as a predictive biomarker for proteasome-inhibitor sensitivity and cannot yet guide clinical treatment choices. Notably, accumulating studies have highlighted a critical role of SKP2 in the development of MM drug resistance. Its abnormal overexpression not only drives disease progression but also mediates resistance to proteasome inhibitors and other anti-myeloma therapies, making SKP2 a potential therapeutic target for overcoming treatment resistance in MM [20,57,61].

Beyond cell-cycle regulation, SKP2 also remodels metabolic phenotypes in MM to sustain malignant growth. The SKP2-LKB1 regulatory axis facilitates the Warburg effect in MM cells via SKP2-mediated LKB1 degradation, which provides sufficient energy support for tumor cell proliferation. Targeting this signaling axis can suppress glycolytic metabolism in MM cells and amplify the antitumor therapeutic efficacy of standard conventional MM therapies [33].

Our group has previously identified a novel regulatory mechanism underlying SKP2-dependent drug resistance in MM. We demonstrated that phosphorylated MARCKS (pMARCKS) acts as an E2F1 cofactor to transcriptionally activate SKP2 expression in MM [61]. Elevated SKP2 subsequently triggers ubiquitination and degradation of p27^Kip1^, abrogating bortezomib-induced G1-phase cell cycle arrest and apoptosis, thereby conferring bortezomib resistance in myeloma cells. Recent mechanistic investigations from our research group have provided key mechanistic insights into how SKP2 contributes to drug resistance in MM. We found that SKP2 is upregulated during MM progression, from pre-myeloma to relapsed stages [17]. Pharmacological inhibition of SKP2 by SkpinC1 reduces STAT3-mediated inflammatory signaling as well as c-MYC and c-MAF expression, impairs MM cell growth, induces apoptosis, and synergistically enhances cell sensitivity to bortezomib. These observations highlight SKP2 inhibition as a promising strategy to overcome MM drug resistance [17,21].

Consistent with these findings, targeted repression of pMARCKS efficiently downregulates SKP2 signaling, reverses p27 depletion and Cyclin E/CDK2 hyperactivation, and restores the susceptibility of resistant MM cells to bortezomib and other anti-myeloma agents. Moreover, stable MARCKS knockdown attenuates oncogenic SKP2 signaling, potently inhibits in vivo myeloma tumor growth, and reverses acquired bortezomib resistance. Collectively, these preclinical findings solidify the SKP2 pathway as a promising therapeutic target for overcoming treatment resistance in relapsed/refractory MM [61].

### 3.7. NK/T-Cell Lymphoma

Extranodal NK/T-cell lymphoma (ENKTL), predominantly nasal-type, is a highly aggressive subtype of non-Hodgkin lymphoma with poor prognosis and limited effective therapeutic regimens. Aberrant SKP2 expression is tightly linked to oncogenic progression and clinical outcomes in nasal-type ENKTL. Immunohistochemical analysis of 39 ENKTL tissue specimens revealed frequent SKP2 overexpression in tumor tissues. Clinically, patients with high SKP2 expression exhibited significantly lower complete remission (CR) rates, increased chemoresistance and shortened median overall survival (OS). Multivariate Cox regression identified elevated SKP2 as an independent adverse prognostic biomarker for OS in ENKTL patients [18].

Another independent tissue profiling study of 48 nasal-type ENKTL cases further demonstrated that SKP2 upregulation is significantly correlated with EBER-positive EBV latent infection, which is the core pathogenic driver of ENKTL [19]. A characteristic SKP2^high^/p27^Kip1low^ immunophenotype was enriched in EBV-positive tumors, with a significant inverse correlation between SKP2 and p27^Kip1^ protein levels [19]. This reciprocal expression pattern supports the EBV-SKP2-p27 regulatory cascade in ENKTL, whereby EBV latent proteins potentiate SCF^Skp2^-mediated ubiquitination and degradation of the cell-cycle suppressor p27 [53]. Notably, this SKP2^high^/p27^low^ expression signature was retrospectively associated with substantially worse overall survival in this cohort, suggesting its potential as an investigational prognostic risk factor [19]. Prospective studies are needed to confirm its clinical utility. Preclinical mechanistic studies and pan-hematological malignancy reviews further indicate SKP2 as a biologically relevant therapeutic target, with small-molecule SKP2 inhibitors capable of reversing chemotherapy resistance and suppressing proliferative signaling in EBV-driven NK/T lymphoma cells [18,19,59].

Epstein–Barr virus (EBV) infection acts as an important oncogenic driver in ENKTL. EBV latent membrane protein 1 (LMP1) and nuclear antigen 3C (EBNA3C) trigger the activation of NF-κB signaling [62]. Activated NF-κB transcriptionally upregulates SKP2 expression. In turn, SKP2 promotes AKT activation by inducing K63-linked ubiquitination of AKT [38]. The activated AKT further sustains persistent NF-κB activity. This establishes a malignant positive feedback circuit of EBV-NF-κB-SKP2-AKT, which amplifies oncogenic signaling and ultimately drives malignant progression [34,38,62].

In addition, several studies demonstrate that the EBV-encoded nuclear antigen EBNA3C physically interacts with the SCF^Skp2^ complex [53]. This interaction activates SCF^Skp2^, which promotes ubiquitination and degradation of p27^Kip1^ and reduces p53 protein levels. Consequently, cell cycle arrest and apoptosis are suppressed, facilitating EBV-mediated cell transformation [53].

## 4. Therapeutic Strategies for Hematological Malignancies Targeting SKP2

### 4.1. Small-Molecule Inhibitors

Substantial preclinical efforts have been dedicated to developing SKP2-targeted small-molecule inhibitors that function through two primary mechanisms: disrupting the binding between SKP2 and its downstream substrates or repressing the E3 ubiquitin ligase catalytic activity of the SCF–SKP2 complex.

Unfortunately, early-generation SKP2 small-molecule inhibitors, including SZL P1–41 and compound C1, are limited by poor pharmacokinetic properties such as low aqueous solubility and inadequate in vivo bioavailability, restricting their clinical translation. However, these early candidates have demonstrated tangible antitumor potency: in vitro assays confirmed their capacity to curb cell proliferation and trigger apoptosis across multiple hematological malignant cell lines [33,63]. In CML xenograft models, co-administration of SKP2-targeted agents with imatinib significantly extended survival, verifying the synergistic therapeutic value of this combinatorial regimen [64]. Likewise, pairing SZL P1-41 with doxorubicin elevated chemotherapy responsiveness and mitigated drug resistance in B-cell precursor acute lymphoblastic leukemia (BCP-ALL) cells [52].

Encouragingly, successive iterative optimization has yielded next-generation SKP2 inhibitors to overcome the aforementioned limitations. The newly identified SKP2-IN-4 achieves stronger growth suppression against NCI-H1299 lung cancer cells and KYSE-510 esophageal cancer cells, accompanied by dramatically lower off-target liabilities relative to earlier chemical scaffolds [65]. This optimized agent exhibits potent anti-proliferative activity in solid-tumor preclinical models. Its activity against hematologic malignancies including AML remains to be experimentally evaluated. Thus, SKP2-IN-4 marks meaningful progress and paves a viable path toward potential future clinical translation.

### 4.2. Molecular Glue Degraders and PROTACs

The development of protein degrader technology (including molecular glues and PROTACs) has provided an innovative strategy for targeting the “undruggable” target SKP2 [4,64]. The intrinsic structural features of SKP2, including a flat and featureless protein–protein interaction (PPI) interface and the lack of druggable deep-binding, ligandable pockets, render it refractory to conventional small-molecule inhibition, which has long hindered targeted drug development [64].

Importantly, multiple emerging mechanistic and structural insights have recently overcome these critical obstacles and greatly advanced the discovery and optimization of SKP2-targeted degraders. Chu et al. identified the first SKP2-targeted molecular glue degrader SKPer1 via their self-developed DEFUSE (DEath FUSion Escaper) high-throughput imaging screening platform [23]. Mechanistically, SKPer1 stabilizes a ternary complex formed by SKP2 and the E3 ubiquitin ligase STUB1 (STIP1 homology and U-box containing protein 1), driving SKP2 ubiquitination and subsequent proteasomal clearance [23]. SKPer1 exclusively eliminates SKP2-overexpressing hematological malignant cells (including AML and DLBCL) while exerting negligible cytotoxicity against normal hematopoietic stem cells. In AML and DLBCL xenograft mouse models, SKPer1 exhibits robust in vivo antitumor activity, favorable pharmacokinetic profiles and satisfactory safety, demonstrating molecular glue degraders as a unique and promising therapeutic branch for SKP2-driven malignancies [23].

In parallel to molecular glue degraders, a natural product-derived universal PROTAC design paradigm for SKP2 ablation has been established [24,36]. By conjugating a hederagenin-based ligand occupying the substrate-recognition LRR domain of SKP2 with CRBN/VHL-recruiting E3 ligase warheads, a series of SKP2-degrading PROTAC candidates exemplified by HD15 have been synthesized [24]. Unlike occupancy-only small-molecule SKP2 inhibitors, HD15 exhibits superior tumor tissue permeability and sustained target depletion, potently suppressing proliferative progression in T-cell acute lymphoblastic leukemia xenograft models [24]. Structural crystallography and cryo-EM investigations have additionally resolved cryptic ligandable pockets within the SKP2-CKS1 binary complex, delivering atomic-level templates for the rational design of high-specificity SKP2 modulators and degraders [64]. Current preclinical observations indicate that SKP2-targeted degraders are associated with a generally acceptable safety profile. Nevertheless, systematic toxicology investigations, especially long-term chronic toxicity studies, remain absent from the published literature. Cumulative preclinical pharmacology and safety data support a favorable therapeutic index that warrants further translational development of SKP2 degraders.

Molecular glue degraders exemplified by SKPer1 and bifunctional PROTACs represented by HD15 circumvent the inherent limitations of traditional occupancy-only SKP2 small-molecule inhibitors. Structural advances resolving cryptic pockets within the SKP2-CKS1 complex, alongside high-throughput screening and modular PROTAC design strategies, fully validate the drugability of SKP2, laying a solid structural and preclinical foundation for the clinical translation of SKP2-targeted protein degradation therapeutics [4,64].

### 4.3. Combination Therapy Strategies

Due to the heterogeneity and complexity of hematological malignancies, single-agent targeted therapy often fails to achieve satisfactory efficacy, necessitating combination therapy to improve outcomes. In recent years, growing evidence has demonstrated that pharmacological inhibition of SKP2 combined with chemotherapies or targeted therapies exerts synergistic anti-leukemic and anti-myeloma effects.

In CML, the SKP2 small-molecule inhibitor SZL P1-41 synergizes with imatinib to suppress cell proliferation and induce apoptosis [51]. Notably, Chen et al. showed that genetic knockdown of SKP2 also enhances the sensitivity of CML cells to imatinib, supporting the potential of SKP2 as a target for overcoming TKI resistance [51].

In BCP-ALL, pharmacological inhibition of SKP2 enhances doxorubicin-induced cytotoxicity and reduces drug resistance in BCP-ALL cell lines by restoring p21^(Cip1/Waf1)^ expression and enhancing DNA damage responses [52].

In MM, the SKP2 inhibitor DT204, identified by Malek et al., enhanced the efficacy of bortezomib in MM cell lines and primary patient cells, overcoming bortezomib resistance through the accumulation of the SKP2 substrate p27 [20]. Consistent with this therapeutic rationale, our group’s preclinical investigation systematically corroborated SKP2 as a master regulator of multidrug resistance in MM. We demonstrated that pharmacological suppression of SKP2 effectively sensitized myeloma cells to standard first-line therapeutics and eliminated acquired drug resistance, further reinforcing the translational value of SKP2-targeted intervention for overcoming treatment refractoriness in MM [17]. These studies indicate that pharmacological SKP2 inhibition combined with conventional therapies improves the therapeutic efficacy by inducing cell cycle arrest, promoting apoptosis, and reversing drug resistance in hematological malignancies [17,20,21,36,51].

In a pan-cancer landscape, Liao et al. established SKP2 as a universal suppressor of anti-tumor immunity and a viable co-target to boost immunotherapy efficacy, which provides theoretical support for exploring SKP2-immunotherapy combinatorial regimens in hematological malignancies [32].

**Figure 2:**
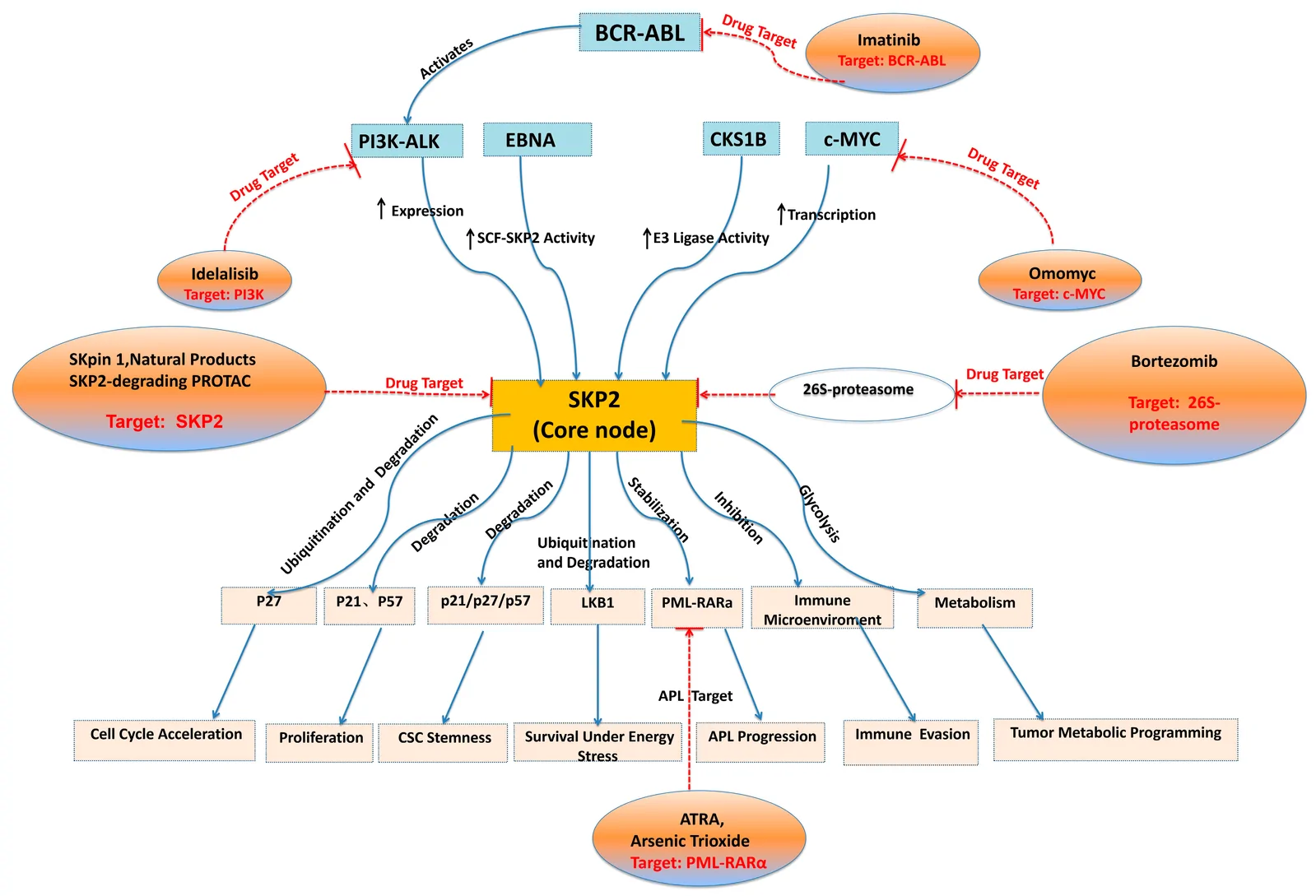
SKP2-centered signaling network and therapeutic interventions in hematological malignancies. Schematic overview of major upstream regulators, downstream substrates, key biological outputs, and therapeutic interventions surrounding SKP2 in hematological malignancies. Selected pharmacological agents targeting distinct signaling nodes are annotated in the diagram. Blue solid arrows represent diverse functional outputs upstream and downstream of SKP2, including substrate ubiquitination-mediated degradation, target-protein stabilization, promotion or inhibition of biological processes. Red dashed arrows denote the targeting effects of agents toward their direct molecular targets. Of note, Omomyc, Idelalisib, ATRA, and Arsenic trioxide serve as illustrative examples with reported biological or clinical activities in hematological malignancies, and these agents are not further elaborated in the main text of this review.

To systematically summarize the preclinical progress of SKP2-targeted therapeutic strategies mentioned above, the key agents, mechanisms, applicable models, and core findings are collated in Table 3, and a schematic overview of these strategies is illustrated in Figure 2.

### 4.4. Clinical Trials Directly or Indirectly Targeting SKP2

Although several selective SKP2 inhibitors remain under preclinical investigation, a limited number of clinical studies have evaluated agents that may modulate SKP2 activity either directly or indirectly (Table 4). Importantly, these clinical studies do not constitute the direct clinical validation of SKP2 as a therapeutic target, as some agents affect broader ubiquitin–proteasome or neddylation pathways.

Betulinic acid has been investigated as a potential direct modulator of SKP2 based on preclinical evidence showing the disruption of the SKP1–SKP2 interaction and subsequent SKP2 degradation [66,67]. Clinically, betulinic acid was evaluated as a topical 20% formulation in a Phase I/II study for dysplastic nevi (NCT00346502). However, the trial was subsequently withdrawn and no participants were enrolled [66]. Thus, clinical evidence supporting the SKP2-directed activity of betulinic acid remains unavailable.

Indirect modulation of the SCF–SKP2 pathway has also been investigated through inhibition of protein neddylation. Pevonedistat (MLN4924), an inhibitor of NEDD8-activating enzyme (NAE), disrupts neddylation-dependent activation of cullin-RING E3 ubiquitin ligases, including SCF complexes containing SKP2 [68,69]. Pevonedistat has progressed into multiple clinical studies, including the Phase 3 PANTHER trial (NCT03268954) in patients with higher-risk myelodysplastic syndromes (MDS), chronic myelomonocytic leukemia (CMML), and low-blast acute myeloid leukemia (AML) [70]. However, the PANTHER trial did not meet its primary endpoint of event-free survival in the intent-to-treat population or in the higher-risk MDS cohort [70]. Although post hoc analyses suggested potential overall survival benefits in selected patients with higher-risk MDS receiving prolonged treatment, these findings do not establish SKP2-specific clinical efficacy [70].

Pevonedistat has also been investigated in combination with irinotecan and temozolomide in a Phase I study of pediatric patients with recurrent or refractory solid tumors and lymphoma (NCT03323034) [71]. Collectively, these studies demonstrate that pharmacological modulation of pathways involving SKP2 has entered clinical investigation. However, the available clinical evidence remains indirect and does not yet establish SKP2 as a clinically validated therapeutic target. Further development of highly selective SKP2-directed agents and biomarker-guided clinical studies will be important to determine whether the promising preclinical therapeutic potential of SKP2 can be translated into clinical benefit.

**Table 4:** Clinical Trials Directly or Indirectly Targeting SKP2-Associated Axis.

Agent	Clinical Trial ID	Phase/Status	Disease Indication	Mechanism	References
Betulinic Acid	NCT00346502	Phase I/II, Withdrawn	Dysplastic nevi	Proposed direct SKP2 modulation based on preclinical evidence	[66]
Pevonedistat (MLN4924)	NCT03268954	Phase III, Completed	HR-MDS, CMML, AML	Indirect modulation through NAE inhibition and disruption of neddylation-dependent SCF activity	[70]
	NCT03323034	Phase I, Completed	Pediatric solid tumors, lymphoma	Indirect pathway modulation through NAE inhibition	[71]

## 5. Conclusions and Future Prospectives

SKP2 and its substrate axis play a central oncogenic role in the initiation, maintenance, and progression of hematological malignancies by regulating multiple biological processes, including cell cycle progression, apoptosis, metabolic reprogramming, and cancer stem cell stemness [25,31,32,33]. In recent years, breakthroughs in the research and development of novel targeted agents including molecular glue degraders and PROTACs have provided a feasible therapeutic strategy for SKP2-targeted therapy. Retrospective clinical data indicate that SKP2 may represent an adverse prognostic marker and a promising therapeutic target for further development in hematological malignancies. The SKP2/p27 expression ratio and SKP2 intracellular localization show potential for risk-stratification purposes, but these remain investigational biomarkers that require large-scale prospective validation before they can be implemented to guide individualized patient treatment [33,42]. Notably, novel molecular glue degraders represented by SKPer1 (XMU-MP-8) are promising candidate agents for precision therapy against hematological malignancies [23].

Despite encouraging preclinical findings, multiple critical barriers hinder the clinical translation of SKP2-targeted therapeutics [36]. Hematological malignancy heterogeneity leads to variable SKP2 expression, making it difficult to screen patients who are responsive to SKP2-targeted therapy. Second, further optimization of the pharmacokinetic properties of SKP2-targeted agents is required to minimize toxic side effects. To mitigate these limitations, the rational design of combination regimens is highly recommended. Large-scale preclinical and clinical data are required to select optimal drug combinations, define administration schedules and determine proper dosages.

Future studies should focus on the following aspects. First, an in-depth exploration of new substrates and non-ubiquitination functions of SKP2 in hematological malignancies is needed to better define its oncogenic network, which can be done through IP-MS screens and ubiquitinome profiling. Second, the development of more efficient and specific SKP2-targeted drugs is essential, with particular emphasis on optimizing the design and delivery systems of molecular glues and PROTACs. Third, preclinical investigation into the potential clinical application of SKP2 as a diagnostic and prognostic marker should be explored further. Overall, these advances position SKP2 as a critical breakthrough in basic and translational research of hematological malignancies, which will help improve patient prognosis.

## Figures and Tables

**Table 1:** Classification of SKP2-Interacting Molecules and Their Functions.

Mode of SKP2-Associated Interaction Category	Molecule	Main Functions	References
Direct proteolytic substrates	p27^Kip1^	Inhibits CDK activity, blocks G1/S transition	[12,13,22]
	p21^(Cip1/Waf1)^	Cell cycle arrest, DNA damage response	[22,29]
	p57^Kip2^	Inhibits cell proliferation, regulates differentiation	[35]
	Cyclin D, Cyclin E	Promote G1-to-S phase transition	[10,11]
	E2F1	Transactivates S-phase genes	[33]
	c-MYC	Drives proliferation and metabolic reprogramming	[17,34,36]
	C/EBPα	Key transcription factor for myeloid differentiation	[37]
Non-proteolytic ubiquitination target	Akt	Promotes cell survival, proliferation, and metabolism; modified via K63-linked ubiquitination without degradation	[33,38]
	RIP1	Regulates apoptosis, necroptosis, and differentiation	[33]
Functional binding partners (Not a direct ubiquitination/degradation substrate of SKP2)	PML-RARα	Oncogenic fusion protein in APL. SKP2 physically binds and stabilizes this fusion protein rather than promoting its degradation	[39]
Upstream regulator	BCR-ABL	Oncogenic fusion kinase in CML; transcriptionally upregulates SKP2 expression via PI3K/Akt/CREB pathway	[40,41]

**Table 2:** Roles of SKP2 in Hematological Malignancies.

Hematological Malignancy	Major Mechanisms of SKP2	Biological Phenotypes	References
Acute Myeloid Leukemia (AML, including APL)	Degrades p27^Kip1^, p21^(Cip1/Waf1)^ and p57^Kip2^, Stabilizes PML-RARα and inhibits its degradation	↑ Proliferation, differentiation block, ↓ Apoptosis, maintenance of leukemic stem-cell properties, ↑ Drug resistance	[14,34,37,39]
Chronic Myeloid Leukemia (CML)	BCR-ABL transcriptionally upregulates SKP2, leading to p27^Kip1^ degradation	↑ Proliferation, TKI resistance	[41,48,51]
B-cell Acute Lymphoblastic Leukemia (B-ALL)	Hyperactivated PI3K/AKT signaling drives SKP2 overexpression; Degrades p21^(Cip1/Waf1)^ and p27^Kip1^	↑ Proliferation, ↑ Drug resistance	[52,53]
T-cell Acute Lymphoblastic Leukemia (T-ALL)	Notch signaling increases SKP2, reduces p27^Kip1^	Abnormal malignant cell proliferation	[54,55]
B-cell lymphoma (DLBCL, representative subtype)	Elevated SKP2 expression drives oncogenic MYC-dependent signaling	↑ Tumor aggressiveness, Shortened overall survival	[15,56]
Multiple Myeloma (MM)	Degrades p27^Kip1^ and LKB1; other tumor suppressors	↑ Proliferation, metabolic reprogramming, ↑ Bortezomib-drug resistance	[17,21,33,57]
NK/T-cell lymphoma (ENKTL, nasal type)	EBV-driven NF-κB signaling upregulates SKP2; SKP2 triggers K63-linked ubiquitination of AKT and promotes p27^Kip1^ degradation	↑ Tumor aggressiveness, chemoresistance, inferior overall survival	[18,19,38]

**Table 3:** Preclinical Development of SKP2-Targeted Therapeutic Strategies in Hematological Malignancies.

Agent/Strategy	Strategy Category	Mechanism	Models	Key Findings	References
SZL P1-41	Small-molecule inhibitor	Blocks SKP2–CKS1 protein–protein interaction, impairing SKP2-mediated substrate recognition	CML, BCP-ALL	Accumulates p27/p21; enhances sensitivity to TKIs and chemotherapy	[51,52]
SKPin C1	Small-molecule inhibitor	Direct SKP2 inhibition	MM (in vitro)	Induces apoptosis	[21]
DT204	SCF complex inhibitor	Disrupts SKP2–CUL1	MM	Overcomes Bortezomib (BTZ) resistance	[20]
SKPer1	Molecular glue degrader	Recruits STUB1 to induce SKP2 degradation	AML, DLBCL	Selective killing of SKP2-high cells, strong in vivo efficacy	[23]
HD15	PROTAC degrader	Targeted proteasomal degradation of SKP2	T-ALL	Effective SKP2 degradation, suppressed T-ALL cell proliferation	[24]
Combination strategies	Rational combinations	SKP2 inhibition combined with proteasome inhibitor (PI), TKI or chemotherapy	CML, MM	Synergistic efficacy, suppression of resistant subclones	[17,51]

## Data Availability

No new data were created or analyzed in this study. Data sharing is not applicable to this article.
